# Exosomal NAMPT from Engineered Mesenchymal Stem Cells Mitigates Aortic Stenosis via Metabolic and Anti-Inflammatory Pathways

**DOI:** 10.3390/ijms27010256

**Published:** 2025-12-25

**Authors:** Dipan Kumar Kundu, Matthew Kiedrowski, James Gadd, Min Gao, Madeline Evan, Yang Wang, Liya Yin, Vahagn Ohanyan, William M. Chilian, Feng Dong

**Affiliations:** 1 Department of Biomedical Sciences, Northeast Ohio Medical University, Rootstown, OH 44272, USA; dkundu@neomed.edu (D.K.K.);; 2 Department of Biomedical Sciences, Kent State University, Kent, OH 44240, USA; 3 Advanced Materials and Liquid Crystal Institute, Kent State University, Kent, OH 44240, USA; 4 College of Medicine, University of Arizona, Tucson, AZ 85721, USA

**Keywords:** NAMPT, endothelial dysfunction, extracellular vesicles, aortic stenosis, cardiovascular regeneration, valve calcification, inflammation, fibrosis, cell-free therapy, metabolic modulation

## Abstract

The aim of this study was to determine whether exosomes from Nicotinamide phosphoribosyltransferase (NAMPT)-overexpressing mesenchymal stem cells (MSC NAMPT-Exo) can attenuate aortic stenosis (AS) and explored the underlying mechanism. NAMPT expression was examined in EC CXCR4 KO (AS) mouse hearts. Six-week-old AS mice received weekly injections of NAMPT-Exo, MSC-Exo, or PBS for three weeks, followed by echocardiography and histological examination of the valves (H&E, Alizarin Red, immunofluorescence). Cardiac ECs from control, AS, and NAMPT-Exo-treated mice were analyzed for miRNA expression (miR-146a-3p/5p, miR-125b-5p, miR-142a-5p). NAMPT expression was decreased in AS hearts. Treatment with NAMPT-Exo reduced aortic valve peak velocity, valvular thickening, and microcalcifications, while improving ejection fraction, fractional shortening, and ventricular dimensions. AS endothelial cells showed elevated levels of miR-146a-3p, miR-146a-5p, and miR-142a-5p, NAMPT-Exo specifically normalized miR-146a-3p. Histology revealed EndMT in AS valves, which was diminished by NAMPT-Exo. In vitro, inhibiting miR-146a-3p suppressed TGF-β-induced EndMT. Our results demonstrate that NAMPT-enriched MSC-derived exosomes effectively slow the progression of AS. Additionally, our findings highlight miR-146a-3p as a key regulator of EndMT, suggesting it as a potential molecular target for future therapies.

## 1. Introduction

### 1.1. Background

Aortic valve stenosis, also known as aortic stenosis (AS), is the most prevalent heart valve disease worldwide, and its incidence increases with age. It is characterized by progressive fibro-calcific remodeling and thickening of the aortic valve cusps, ultimately leading to left ventricular outflow obstruction. Among adults aged 75 years and older in North America and Europe, AS affects an estimated 7.6 million individuals [1]. The pathophysiology of AS is complex and develops through a chronic process with two distinct phases [2,3]. The initiation phase is characterized by endothelial injury and lipid infiltration, which trigger inflammation and activate valvular interstitial cells. In the later propagation phase, these cells undergo osteogenic reprogramming, leading to progressive calcification and fibrotic thickening of the valve leaflets. This structural remodeling narrows the aortic valve opening, increases left ventricular pressure overload, and ultimately results in heart failure if left untreated. A major challenge in the field is the absence of effective pharmacological therapy capable of slowing or halting disease progression. Valve replacement remains the only definitive treatment, and untreated symptomatic AS is associated with high morbidity and mortality [1,2]. This therapeutic gap underscores the urgent need for novel therapeutic strategies targeting the underlying disease mechanisms.

### 1.2. NAD^+^ Metabolism and NAMPT in Cardiovascular Disease

Nicotinamide phosphoribosyltransferase (NAMPT) is a key regulator of cellular homeostasis as the rate-limiting enzyme in the NAD^+^ salvage pathway, maintaining intracellular NAD^+^ pools required for energy metabolism, mitochondrial function, redox balance, DNA repair, and epigenetic regulation [4,5,6,7]. NAD^+^ levels decline with major disease-associated risk factors, including age, obesity, and hypertension, and NAD^+^ replenishment has demonstrated therapeutic benefits in multiple preclinical disease models [5,8,9]. Declines in NAMPT expression correspondingly reduce NAD^+^ levels, suggesting potential therapeutic implications for NAMPT supplementation [10]. In addition to its intracellular function, extracellular NAMPT (eNAMPT), particularly when packaged within extracellular vesicles (EVs), can enhance NAD^+^ biosynthesis in recipient cells and contribute to systemic metabolic communication. For example, eNAMPT-enriched EVs derived from young adipocytes were able to improve cellular function and lifespan in aged mice, suggesting an important role for EV-contained eNAMPT in maintaining inter-tissue NAD^+^ homeostasis and cellular health [7]. Within the cardiovascular system, NAMPT signaling has been linked to cardioprotection through anti-inflammatory, anti-apoptotic, and antioxidant actions predominantly via NAD^+^-dependent and sirtuin-mediated pathways [11,12,13,14]. Dysregulation or loss of NAMPT activity, in contrast, has been associated with impaired NAD+ metabolism, increased oxidative stress, endothelial dysfunction, and adverse cardiovascular remodeling [15,16]. Collectively, these findings highlight NAMPT as a protective mediator in cardiovascular pathophysiology.

### 1.3. Therapeutic Potential of MSC-Derived Exosomes

Mesenchymal stem cells (MSCs) have been widely used in regenerative medicine due to their multipotent capacity, immunomodulatory properties, and ability to be expanded and manipulated in vitro. MSCs contribute to cardiovascular repair by promoting angiogenesis, reducing the level of inflammation, decreasing apoptosis, and inhibiting fibrosis [17,18]. Although numerous preclinical studies demonstrate that MSC transplantation improves cardiac repair, challenges such as low migration to the ischemic myocardium, poor tissue retention, immune rejection, and low survival rate after transplantation, limit its clinical application. Increasing evidence indicates that the therapeutic benefits of MSCs are primarily mediated through paracrine mechanisms, particularly via exosomes, which are nanoscale vesicles (50–150 nm) containing bioactive proteins, lipids, and genetic material [19,20]. Exosomes can be selectively taken up by neighboring or distant cells, even those far from their release site, and can modulate the properties or function of the recipient cells by delivering their bioactive compounds [21]. In various disease models, MSC-derived exosomes have been shown to replicate the therapeutic benefits of MSCs themselves [20,22,23]. Compared with direct MSC transplantation, MSC exosomes offer advantages such as enhanced targeting, lower immunogenicity, and improved stability, thereby overcoming major barriers to clinical translation [24,25]. Furthermore, engineering MSC exosomes to enrich their cargo with specific bioactive molecules further enhances their therapeutic efficacy, positioning them as promising candidates for clinical translation [19,21].

### 1.4. Aortic Stenosis Model and Study Rationale

Our group previously established an endothelial cell-specific CXCR4 knockout (EC CXCR4 KO) mouse model, which spontaneously develops AS accompanied by significant functional impairment, including elevated aortic valve peak velocity, increased pressure gradient, ventricular hypertrophy, and reduced ejection fraction [26]. Given the limitations of other AS animal models, such as the lack of spontaneous development and clinically relevant hemodynamic obstruction [27,28], the EC CXCR4 KO mouse offers a robust and reproducible platform to investigate AS pathogenesis and evaluate therapeutic interventions. Despite growing evidence linking endothelial dysfunction, inflammatory microRNAs, and metabolic regulators to aortic valve stenosis (AS), translating these molecular insights into clinically actionable biomarkers or targeted therapies remains limited. The lack of in vivo endothelial-specific mechanistic studies, coupled with insufficient validation in human AS tissues, represents a major translational gap that prevents the development of effective therapeutic strategies. This study was designed to determine whether NAMPT-overexpressing MSC-derived exosomes can serve as a cell-free therapeutic strategy to slow or reverse aortic stenosis progression by targeting metabolic and inflammatory pathways.

## 2. Results

### 2.1. NAMPT Expression Is Decreased in AS Mice

**Figure 1:**
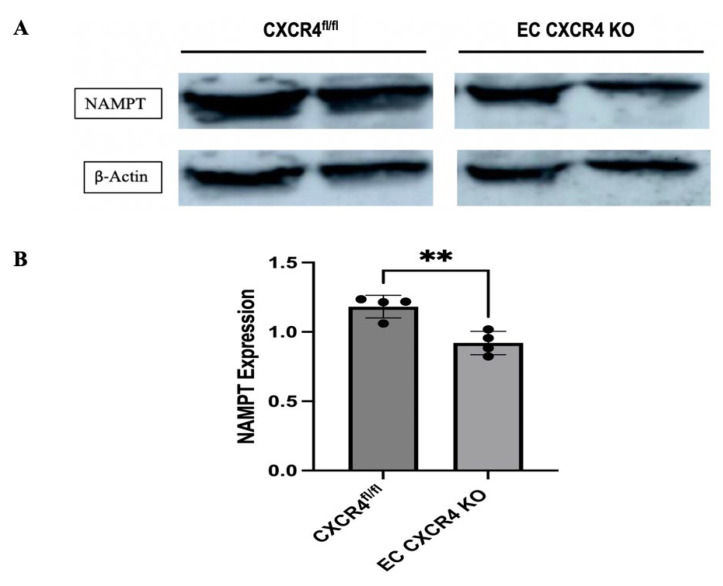
(**A**) Representative Western blot showing NAMPT expression in the heart tissues of CXCR4^fl/fl^ control and EC CXCR4 KO (AS) mice. (**B**) Quantification of NAMPT expression (*n* = 4), expression was normalized to β-actin. Unpaired Student’s *t* test; ** *p* < 0.01 for CXCR4^fl/fl^ vs. EC CXCR4 KO mice. Each black dot represents an individual sample.

We first tested whether there was a difference in NAMPT expression between EC CXCR4 KO (AS) and CXCR4^fl/fl^ control mice. Western blot revealed that NAMPT expression is significantly decreased in the heart tissues of AS mice compared to the control mice (Figure 1A,B).

### 2.2. Characterization of NAMPT-Exo

**Figure 2:**
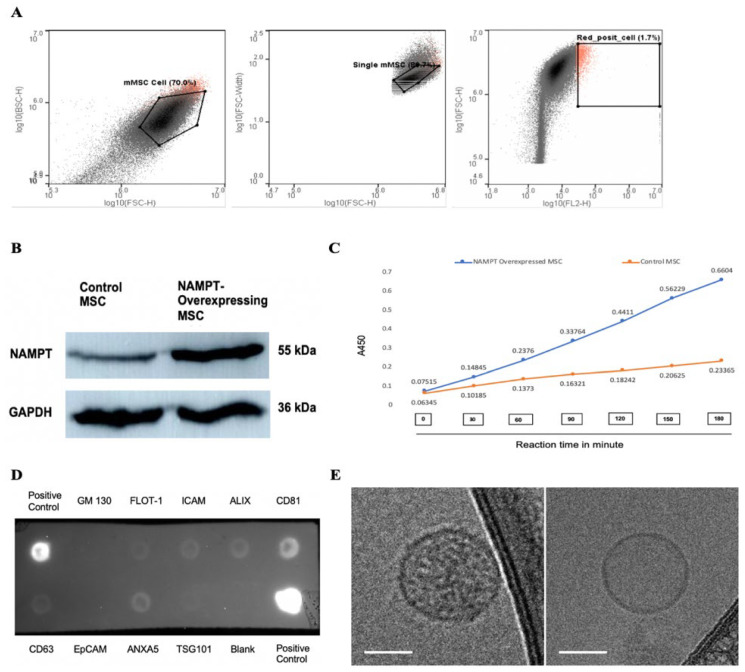
(**A**) Sorting of tdTomato-positive NAMPT-overexpressing MSCs using fluorescence activated cell sorting (FACS). (**B**) Representative Western blot showing NAMPT expression in purified NAMPT-overexpressing MSCs and non-transduced control MSCs. (**C**) NAMPT activity assay in immunoprecipitated lysates from NAMPT-overexpressing MSCs and control MSCs. (**D**) Antibody array assay detecting exosome markers in the isolated exosome sample. (**E**) Representative cryo-TEM images of exosomes; scale bars: 50 nm.

The NAMPT gene was cloned into a lentiviral vector that co-expresses a bright red fluorescent protein, tdTomato, and then transduced into MSCs using a lentiviral expression system. Successful cloning was confirmed by sequencing, and overexpression into MSCs was identified by tdTomato fluorescence microscopy. We further purified tdTomato-positive NAMPT-overexpressing MSCs using a fluorescence-activated cell sorter (FACS) (Figure 2A). NAMPT overexpression in MSCs was confirmed by Western blot and an in vitro bioactivity assay. Western blot analysis demonstrated a ~12-fold increase in NAMPT protein expression in the overexpressing MSCs compared to non-transduced control MSCs (Figure 2B). Using a NAMPT activity assay, we compared NAMPT activity in the immunoprecipitated cell lysates of NAMPT-overexpressed and control MSCs. A significantly higher enzymatic activity in the overexpressed lysate further confirmed the overexpression of NAMPT in MSCs and suggested elevated NAMPT bioactivity in these cells (Figure 2C). Isolated exosomes from control and NAMPT-overexpressing MSCs were assessed by an antibody array, confirming the presence of common exosome markers such as CD63, CD81, TSG101, and Alix (Figure 2D). Cryo-TEM revealed membrane-enclosed vesicles with typical exosome morphology (Figure 2E).

### 2.3. NAMPT-Exo Treatment Attenuates AS Progression

**Figure 3:**
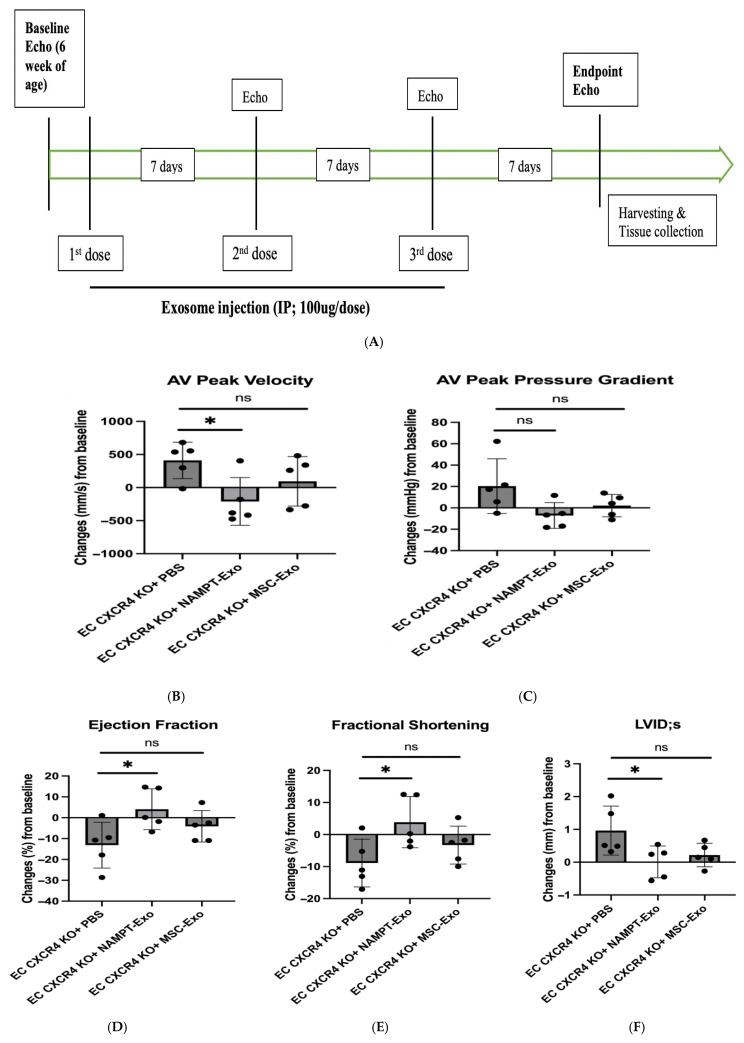

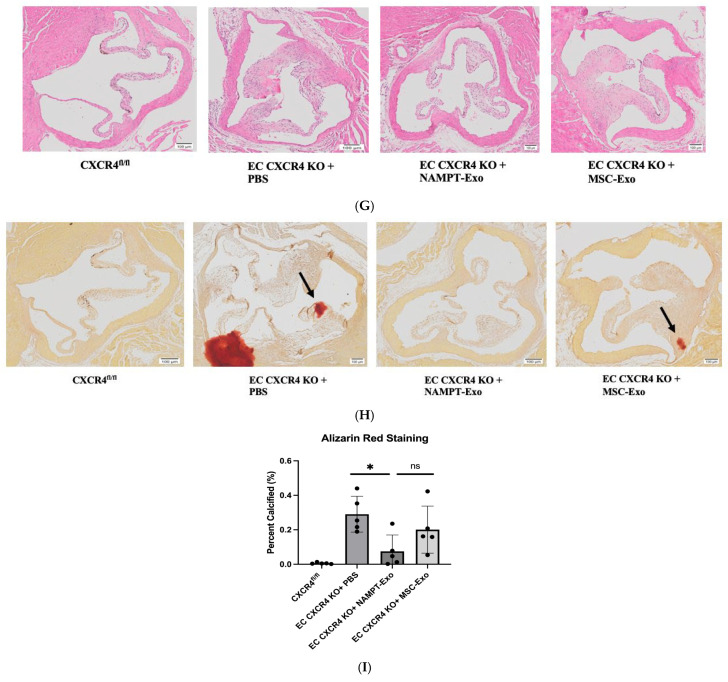
(**A**) Schematic diagram showing the study design. EC CXCR4 KO (AS) mice underwent baseline echocardiography at 6 weeks of age, followed by intraperitoneal (IP) injections of exosomes derived from NAMPT-enriched MSCs or control MSCs at a dose of 100 µg (~1.91 × 10^11^ particles) per injection or vehicle (PBS) for three doses once a week. (**B**–**F**) Echocardiography analysis shows IP injection of NAMPT-Exo improves AS outcome. (**B**) Aortic Valve (AV) peak velocity (mm/s). (**C**) AV peak pressure gradient (mmHg). (**D**) Ejection fractions (%); (**E**) Fractional shortening (%); and (**F**) Left ventricular internal dimension at end-systole (LVIDs) (mm) across treatment groups; *n* = 5, data are mean ± SD, and are represented as the difference between after (endpoint) and before (baseline) treatment in each group, * *p* < 0.05, one-way ANOVA followed by post hoc Dunnett test. (**G**) H&E staining showing general morphology of aortic valves. (**H**) Alizarin red staining for calcification. Arrows indicate positively stained calcified regions. (**I**) Alizarin red staining quantification (*n* = 5). One-way ANOVA followed by post hoc Tukey HSD test; * *p* < 0.05. “ns” represents “not significant”; each black dot represents an individual sample.

Next, to explore the role of NAMPT-Exo in aortic stenosis, we administered three doses of NAMPT-Exo to 6-week-old EC CXCR4 KO AS mice, along with PBS control and MSC-Exo, in different groups once a week (Figure 3A). The exosome dose was selected based on commonly reported exosome doses in mice from previously published studies [19,29]. Mice treated with NAMPT-Exo injection exhibited a significantly reduced aortic valve (AV) peak velocity and showed an improved trend in AV peak pressure gradient compared to the PBS control. (Figure 3B,C). Treatment also improved compromised cardiac function associated with aortic stenosis, including ejection fraction, fractional shortening, and LVID (Figure 3D–F). Consistent with these findings, histological assessment of aortic valve sections 7 days after the last dose of NAMPT-Exo injection (followed by endpoint echocardiography) also showed improvement. Reduced thickening (H&E staining) and calcification (Alizarin red staining) of stenotic valve leaflets were observed in the group treated with NAMPT-Exo injection (Figure 3G–I). These data suggested that NAMPT-Exo treatment halted the progression of aortic stenosis in EC CXCR4 KO mice.

### 2.4. In Vivo Uptake of NAMPT-Exo by Cardiac Tissue

**Figure 4:**
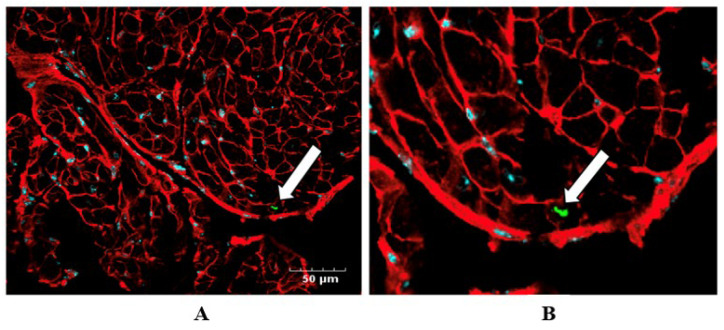
(**A**,**B**) Confocal imaging confirms NAMPT-Exo uptake by cardiac tissue following IP injections. (**A**) Representative immunofluorescence image showing tdTomato (green; detected using a tdTomato-specific antibody conjugated with a green fluorophore), WGA (red; labeling cardiomyocyte membrane), and DAPI (blue, labeling nuclei) in the heart tissue sections. (**B**) Enlarged view highlighting tdTomato-positive staining (white arrow), indicating NAMPT-Exo internalization in cardiac tissue.

To assess in vivo uptake of NAMPT-Exo by cardiomyocytes, we performed confocal microscopy on heart tissue sections from AS mice treated with NAMPT-Exo. Co-expression of NAMPT with tdTomato enabled fluorescent tracking of exogenous NAMPT. Seven days after the final IP injection, hearts were harvested and sectioned for immunofluorescent analysis. Cardiomyocyte membranes were labeled with Wheat Germ Agglutinin (WGA; red), and tdTomato was detected with a tdTomato-specific primary antibody directly conjugated to a green fluorophore. Confocal images revealed tdTomato signal within cardiomyocytes, indicating successful uptake of NAMPT-Exo by cardiac tissue following IP injections (Figure 4).

### 2.5. NAMPT-Exo Suppresses EndMT in the Stenotic Aortic Valve

**Figure 5:**
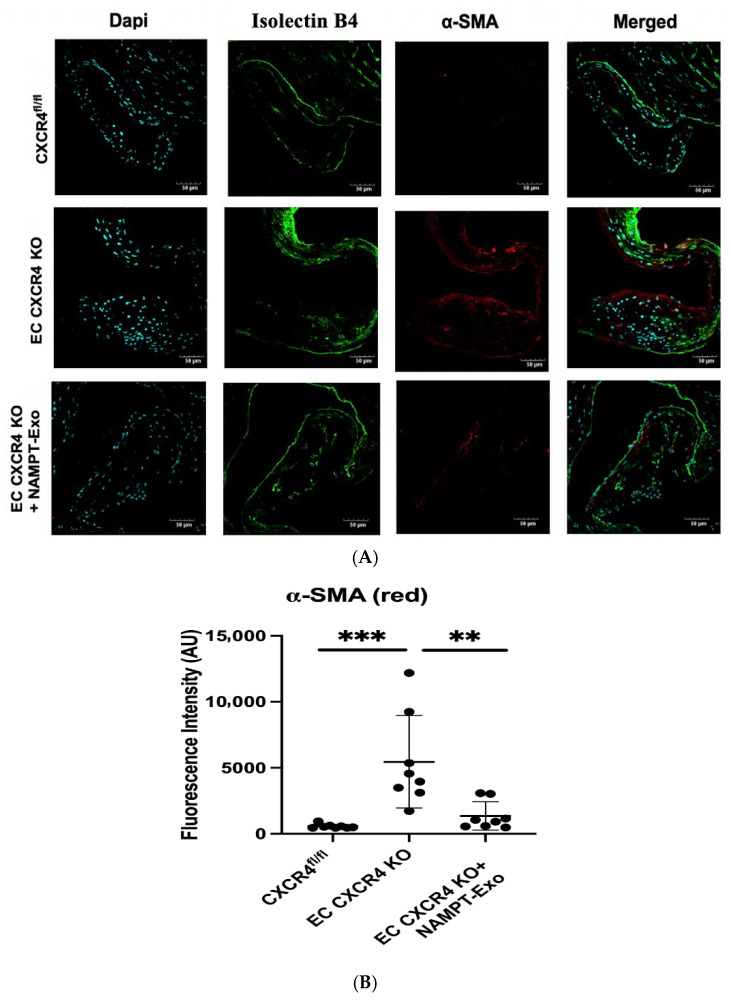
(**A**,**B**) Immunohistochemistry analysis of aortic valve sections. (**A**) Representative images showing co-staining for endothelial marker Isolectin B4 (green) and mesenchymal marker α-SMA (red). DAPI (blue) labels nuclei. Scale bars indicate 50 µm. (**B**) Quantification of fluorescence intensity for α-SMA (*n* = 8). Data are presented as mean ± SD. One-way ANOVA followed by post hoc Tukey HSD test; ** *p* < 0.01, *** *p* < 0.001. α-SMA indicates α-smooth muscle actin; and AU, arbitrary unit. Each black dot represents an individual sample.

Accumulating evidence suggests that endothelial-to-mesenchymal transition (EndMT) plays a significant role in the progression of aortic stenosis [30,31,32]. To determine whether EndMT occurs in the aortic valve leaflets of EC CXCR4 KO mice and to evaluate the effect of NAMPT-Exo treatment on this process, we performed immunofluorescence staining for the endothelial marker Isolectin B4 and the mesenchymal marker α-smooth muscle actin (α-SMA) in aortic valve sections (Figure 5A). In age-matched mice, EC CXCR4 KO groups showed markedly increased α-SMA immunoreactivity compared to CXCR4^fl/fl^ controls, confirming heightened EndMT activity within the stenotic valve (Figure 5B). Conversely, valve sections from NAMPT-Exo-treated EC CXCR4 KO mice showed a significant decrease in α-SMA expression.

### 2.6. NAMPT-Exo Restores miR-146a-3p Levels in AS Mice

Data Preprocessing and Quality Control: RNA samples used for microRNA analysis exhibited consistent purity and integrity across experimental groups, with A260/280 ratios between 1.8 and 2.0 and reliable amplification performance in RT–qPCR assays. No systematic differences in RNA quality or assay performance were observed, supporting the robustness of downstream expression analyses.

**Figure 6:**
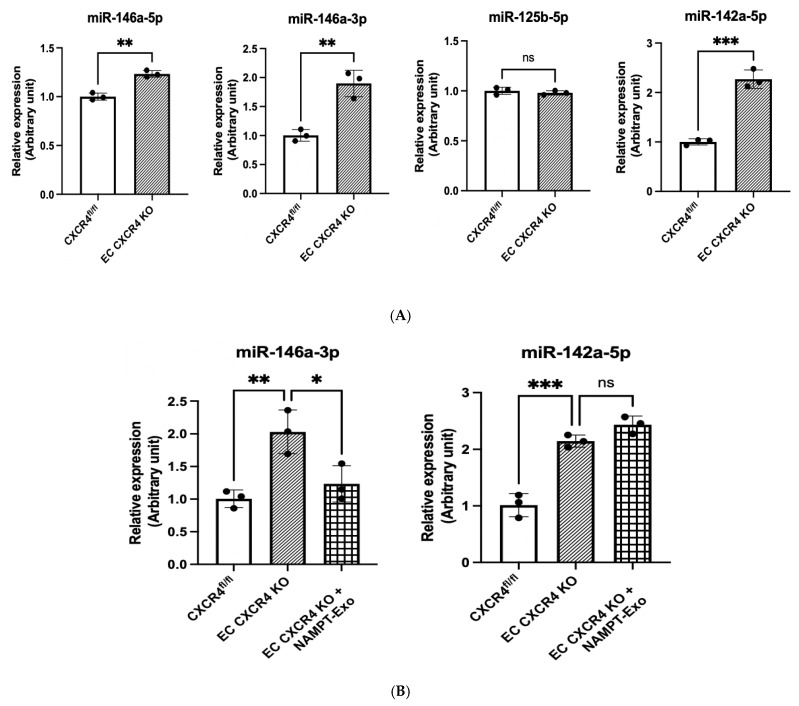
(**A**,**B**) qPCR analysis on the miRNAs’ expression in different groups. (**A**) Relative expression in the cardiac endothelial cell of CXCR4^fl/fl^ vs. EC CXCR4 KO group. (**B**) Changes in relative expression after NAMPT-Exo treatment. *n* = 3; data are mean ± SD. One-way ANOVA followed by post hoc Tukey test; * *p* < 0.05, ** *p* < 0.01, and *** *p* <0.001. “ns” represents “not significant”. Each black dot represents an individual sample.

MicroRNA dysregulation in the aortic valve and surrounding tissues has been linked to the development and progression of aortic stenosis [33,34,35]. We examined the expression of four candidate miRNAs in heart endothelial cells of EC CXCR4 KO (AS) and CXCR4^fl/fl^ control mice: miR-146a-5p, miR-146a-3p, miR-125b-5p, and miR-142a-5p. These miRNAs were selected based on the prior literature implicating them in CXCR4 signaling and endothelial activation, as well as on their consistent dysregulation in AS. We found that miR-146a-3p (1.90-fold increase) and miR-142a-5p (2.27-fold increase) were significantly elevated in the EC CXCR4 KO AS mice compared to controls (Figure 6A). Next, we explored whether reversing dysregulated microRNA expression could explain the benefits of NAMPT-Exo treatment. We compared miR-146a-3p and miR-142a-5p levels in heart endothelial cells of NAMPT-Exo-treated mice, untreated EC CXCR4 KO mice, and CXCR4fl/fl controls. NAMPT-Exo treatment partially restored miR-146a-3p levels to near control levels (relative expression 1.23 vs. 2.01 in untreated EC CXCR4 KO, relative to floxed controls normalized to 1.0). However, miR-142a-5p levels remained unchanged (relative expression 2.44 vs. 2.15 in untreated EC CXCR4 KO, relative to floxed controls normalized to 1.0) (Figure 6B). Based on this, we focused on miR-146a-3p to assess its direct role in stenosis progression.

### 2.7. MiR-146a-3p Inhibition Suppresses EndMT in Cardiac Endothelial Cells from AS Mice

**Figure 7:**
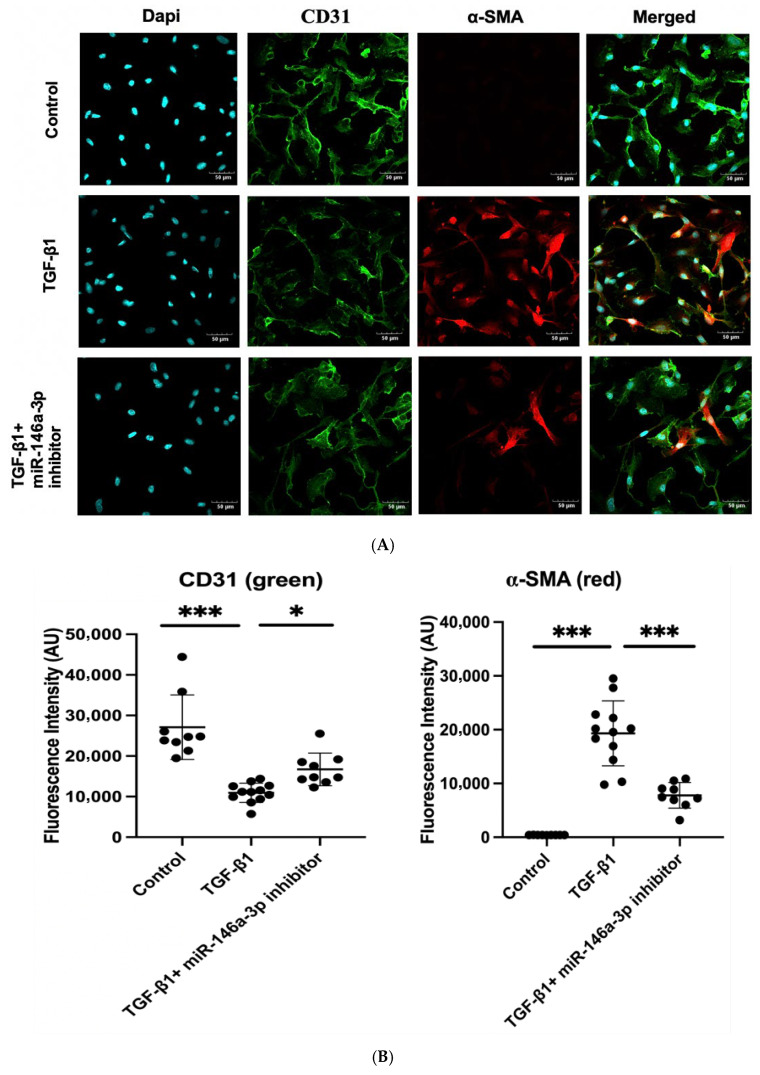

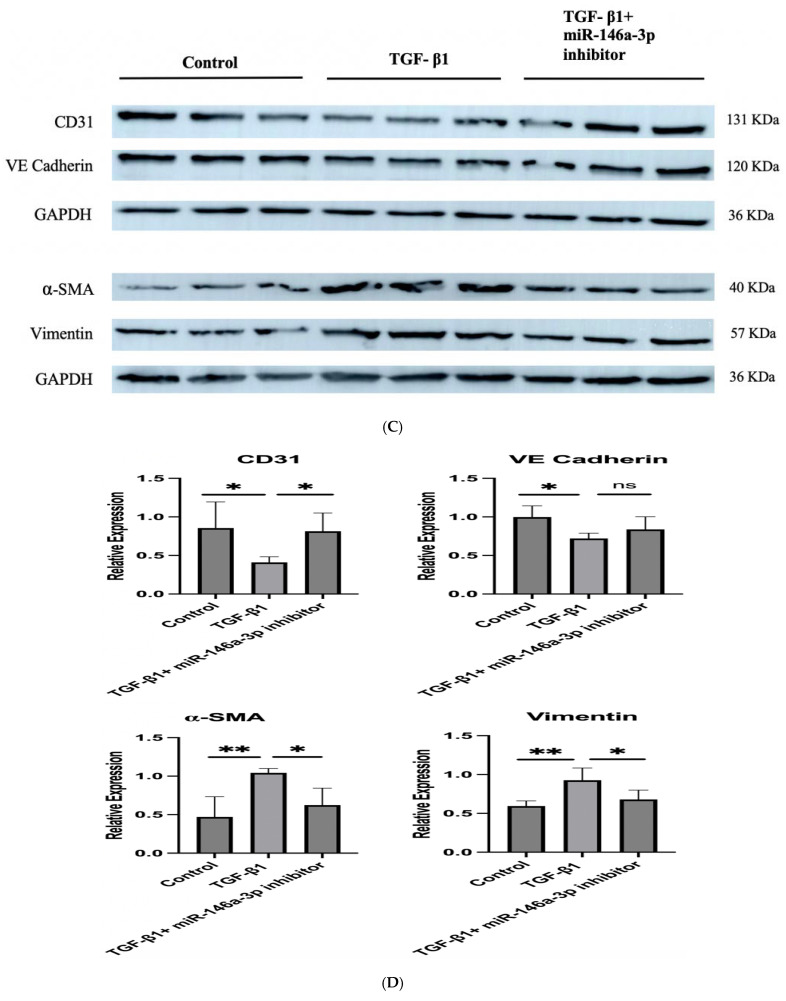
(**A**–**D**) TGF-β1induced EndMT in cardiac endothelial cells from EC CXCR4 KO mice, and miR-146a-3p inhibition suppressed it. (**A**) Representative immunofluorescence images of cardiac endothelial cells labeled with CD31 (green) and α-SMA (red) under different treatment conditions: untreated control, TGF-β1 only, and TGF-β1 with miR-146a-3p inhibitor. Scale bars show 50 µm. (**B**) Quantification of fluorescence intensity for CD31 and α-SMA. *n* = 9–12; Data are mean ± SD. (**C**) Representative Western blot images showing expression of endothelial (CD31 and VE-Cadherin) and mesenchymal markers (alpha-SMA and Vimentin) in different treatment groups. (**D**) Quantification of protein expression in different groups (*n* = 4), expression was normalized to GAPDH. Bars show mean ± SD. For (**B**,**D**), one-way ANOVA followed by post hoc Tukey HSD test; * *p* < 0.05, ** *p* < 0.01, *** *p* < 0.001. “ns” represents “not significant”. TGF-β1 indicates transforming growth factor β1; CD31, cluster of differentiation 31, and alpha-SMA, alpha-smooth muscle actin. Each black dot represents an individual sample.

Since NAMPT-Exo treatment suppressed EndMT in aortic valves and also restored the expression of miR-146a-3p, we next evaluated whether miR-146a-3p directly influences EndMT progression. To investigate this, we used an in vitro model of TGF-β1-induced EndMT in endothelial cells [36,37]. We isolated cardiac endothelial cells from the EC CXCR4 KO mice and exposed them to TGF-β1 with or without the miR-146a-3p inhibitor. The miR-146a-3p inhibitor reduced TGF-β1-induced expression of the mesenchymal marker α-SMA in ECs, as shown by immunofluorescence staining (Figure 7A,B). Western blot analysis of endothelial markers CD31 and VE-cadherin, along with mesenchymal markers α-SMA and Vimentin, further confirmed that inhibiting miR-146a-3p decreased TGF-β1-mediated EndMT induction (Figure 7C,D).

## 3. Discussion

The present study provides new insights into the therapeutic potential of exosome-based treatments for aortic stenosis (AS). Our main findings are: (1) NAMPT-enriched MSC-derived exosomes slow the progression of aortic stenosis and enhance impaired cardiac function associated with AS; (2) these therapeutic benefits are linked to the inhibition of endothelial-to-mesenchymal transition (EndMT) in the aortic valve endothelium; and (3) exosome-mediated regulation of miR-146a-3p is crucial in controlling EndMT during AS development. To our knowledge, this is the first evidence that an exosome-based therapy can reduce the in vivo progression of AS, highlighting a potential new approach for disease modification where none currently exists.

We previously reported that endothelial-specific CXCR4 deletion in mice (EC CXCR4 KO) leads to the development of AS, with disease manifestations appearing as early as 3 weeks of age [26]. Given the downregulation of NAMPT in these AS mice and its known active secretion via exosomes [7,38], we hypothesized that NAMPT-containing MSC exosomes could have a positive impact on these mice. Therefore, we sought to overexpress NAMPT in MSCs to collect NAMPT-enriched MSC exosomes (NAMPT-Exo) for the treatment of AS mice. We treated 6-week-old mice with AS using NAMPT-Exo. Our data demonstrates that NAMPT-Exo therapy effectively attenuates the progression of AS in EC CXCR4 KO mice, a model characterized by aggressive valvular degeneration. Mice receiving NAMPT-Exo exhibited significant improvement in both echocardiographic and histological markers of AS severity compared to PBS- and MSC-Exo-treated controls. Notably, NAMPT-Exo administration reduced aortic valve peak velocity and showed a favorable trend in lowering the peak pressure gradient, indicating mitigation of valvular outflow obstruction. Cardiac function, assessed by ejection fraction, fractional shortening, and left ventricular internal diameter (LVID), also improved, suggesting systemic cardiovascular benefits beyond the valve. Histological analyses further supported these observations, with NAMPT-Exo treatment resulting in reduced leaflet thickening and calcification, key pathological features of AS. Collectively, these data support a protective role for NAMPT-Exo in modulating the progression of AS in the context of CXCR4 deficiency, potentially through anti-inflammatory, anti-calcific, or reparative mechanisms, warranting further mechanistic investigation and therapeutic exploration.

Our results are consistent with emerging evidence supporting the cardioprotective properties of exosome-based therapies. Prior studies have shown that MSC-derived exosomes mimic the benefits of cell therapy, improving cardiac hypertrophy and function in heart failure in animal models [19,39,40]. Furthermore, engineered extracellular vesicles have been successfully deployed to deliver therapeutic cargo directly to calcified aortic valves, demonstrating the feasibility of exosome-based interventions in valvular heart disease [41]. The ability of NAMPT-Exo to improve both functional and structural indices of AS is especially compelling given the absence of effective medical therapies for this condition. Currently, treatment for AS is limited to mechanical valve replacement via surgical or transcatheter approaches, with no pharmacologic agents capable of halting or reversing disease progression [1,2]. Previous trials of lipid-lowering and anti-inflammatory agents have failed to alter the natural history of AS [42,43], highlighting the urgent need for innovative therapeutic strategies. In this context, our findings position NAMPT-enriched exosomes as a promising and novel biologic platform for the treatment of aortic stenosis.

Endothelial-to-mesenchymal transition (EndMT) has emerged as a key contributor to valvular remodeling in AS [30,31,32,44]. Our findings provide compelling evidence that EndMT contributes to the pathogenesis of aortic stenosis (AS) and that NAMPT-Exo therapy mitigates this pathogenic process. Immunofluorescence staining revealed a substantial increase in α-smooth muscle actin (α-SMA), a mesenchymal marker, in the aortic valve leaflets of EC CXCR4 KO (AS) mice compared to their CXCR4^fl/fl^ littermate controls, indicating enhanced EndMT activity within the diseased valves. This shift was accompanied by a loss of CD31, an endothelial marker, further supporting the transition from an endothelial to mesenchymal phenotype. Importantly, NAMPT-Exo treatment significantly reduced α-SMA expression in the valves of AS mice, suggesting that NAMPT delivery via exosomes can suppress EndMT in vivo. These results align with the growing recognition of EndMT as a key contributor to valve fibrosis and calcification in AS and support the notion that targeting EndMT may be a viable therapeutic strategy. By attenuating EndMT, NAMPT-Exo may help preserve endothelial integrity and reduce mesenchymal-driven pathological remodeling of the valve, offering mechanistic insight into its protective effects in this model of aggressive valvular degeneration.

MicroRNAs are increasingly recognized as critical regulators in cardiovascular disease, including calcific valve disease [33,34,35,45,46]. In the context of AS, miR-146a has been implicated in inflammatory and remodeling processes. Patients with calcific AS show elevated miR-146a levels in diseased valves and myocardium, correlating with valvular inflammation and left ventricular hypertrophy [47,48,49]. In our study, we identified a marked dysregulation of miR-146a-3p in the endothelial cells of AS mice, which was largely normalized after NAMPT-Exo treatment. In vitro experiments further demonstrated that inhibition of miR-146a-3p blunted TGF-β-induced EndMT, implicating this microRNA as an upstream regulator of endothelial plasticity in AS. These results extend prior observations on miR-146a’s role in cardiovascular pathology by specifically tying the 3p strand to valvular EndMT. Our data suggest that NAMPT-Exo mitigates EndMT, at least in part, by downregulating miR-146a-3p, thereby dampening TGF-β signaling and limiting endothelial transformation. Thus, miR-146a-3p axis represents a novel therapeutic target, with potential for modulating early upstream events in the progression of calcific AS.

### 3.1. Limitations

This study has several limitations. First, while the therapeutic effects of NAMPT-Exo are largely attributed to NAMPT enrichment, exosomes contain diverse bioactive molecules. Overexpression of NAMPT may have altered the global miRNA and protein content of exosomes, suggesting that other cargo components could contribute to the observed effects. Second, although miR-146a-3p was identified as a regulator of EndMT and validated in vitro, its causal role in vivo remains unproven. Genetic manipulation of miR-146a-3p in EC CXCR4 KO mice, via targeted knockdown or overexpression, would provide stronger evidence linking this microRNA to disease progression and NAMPT-Exo efficacy. Finally, the mechanism by which exosome treatment modulates miR-146a-3p expression is not fully understood. Exosomes may influence gene regulation through multiple mechanisms, including modulation of non-coding RNA networks and epigenetic modifications such as DNA methylation [50,51,52,53].

### 3.2. Future Perspectives

Future studies should address the above limitations and further validate the therapeutic potential of NAMPT-enriched MSC-derived exosomes. Comprehensive omics profiling of NAMPT-Exo versus control exosomes is needed to identify specific molecular mediators responsible for the observed effects. Investigating the causal role of miR-146a-3p in vivo via targeted genetic manipulation will strengthen mechanistic insights. Additionally, elucidating the precise molecular pathways through which exosome cargo modulates gene expression and EndMT will be critical for optimizing exosome-based therapies and translating them into effective interventions for aortic stenosis.

## 4. Materials and Methods

### 4.1. Animals

Endothelial-specific CXCR4 knockout (ES CXCR4 KO) mice were generated as previously reported [26]. Animals were housed in the animal facility of Northeast Ohio Medical University under temperature-controlled conditions, with free access to water and standard rodent chow. All animal procedures were approved by the Institutional Animal Care and Use Committee (IACUC) of Northeast Ohio Medical University and were conducted in accordance with the NIH guidelines (Guide for the Care and Use of Laboratory Animals).

### 4.2. MSC Isolation and Culture

MSCs were isolated from mouse bone marrow and cultured in a standard incubator as previously described [54]. Briefly, six-week-old mice were euthanized, and the hindlimbs were removed. Bone marrow was flushed with flush medium (Alpha Medium with 2 g/L NaHCO_3_, 10% horse serum, 10% fetal bovine serum, 1% l-glutamine, 1% penicillin-streptomycin) from thoroughly cleaned femurs and tibias and passed through a 70-μm cell filter. The collected bone marrow cells were washed and incubated at 37 °C. Nonadherent cells were removed by replacing the medium after 24 h. Once the cells reached 80% confluency, adherent cells were detached and were depleted for CD45^+^ and CD34^+^ cells by negative selection with primary PE-conjugated antibodies (mouse anti-CD34 and mouse anti-CD45 antibodies, BD Biosciences, San Diego, CA, USA) using the EasySep PE selection kit according to the manufacturer’s instructions (Stem Cell Technologies, Vancouver, BC, Canada). The sorted MSCs were replated in MSC medium and were subsequently passaged until passage 3. The cells were passaged when reaching 80–85% confluency, and the culture medium was changed every 2–3 days.

### 4.3. Lentiviral Construction

The backbone vector pLVX-IRES-tdTomato (Takara Bio, San Jose, CA, USA) was used to construct a lentiviral vector containing NAMPT. Mouse NAMPT fragment was amplified from total RNA using primers listed in Table 1.

Restriction sites for BamHI & XhoI were added at the 5′ ends of the forward and reverse primers, respectively. The PCR product and the backbone vector were digested with XhoI and BamHI (Invitrogen, Waltham, MA, USA) and ligated using In-Fusion cloning (Takara Bio, San Jose, CA, USA). The resulting construct was verified by Sanger sequencing. For lentivirus packing, the reconstructed vector containing NAMPT was co-incubated with Lenti-X Packaging Single Shot (Takara Bio, 631278, San Jose, CA, USA) into Lenti-X 293T cells according to the manufacturer’s protocol. Fresh media was added to the cells following overnight incubation. Viral supernatants were collected after 48 h and were filtered through a 0.45 µm cellulose acetate filter. The supernatant was then concentrated using Lenti-X concentrator (Takara Bio, 631231, San Jose, CA, USA) and tittered using Lenti-X GoStix Plus (Takara Bio, 631280, San Jose, CA, USA). The concentrated lentivirus was stored at −80 °C until use.

### 4.4. NAMPT Overexpression in MSC

For transducing MSC, lentiviruses were diluted with medium containing polybrene (5 µg/mL) and were added to the cells. After 24 h, virus-containing transduction medium was replaced with fresh medium, and cells were further incubated for 48 h to isolate the stably transfected cell population. Transduced MSCs were screened by fluorescence microscopy for tdTomato fluorescence, and tdTomato-positive NAMPT-overexpressing MSCs were then purified by fluorescence-activated cell sorting (Wolf Cell sorter, NanoCellect, San Diego, CA, USA). NAMPT protein expression was analyzed by Western blot in control MSC (non-transduced) and NAMPT-overexpressing MSC to confirm the overexpression.

### 4.5. NAMPT Activity Assay

To investigate the effect of NAMPT overexpression on NAMPT activity in the MSCs, an activity assay kit (Abcam, Cambridge, MA, USA ab221819) was used according to the manufacturer’s instructions. Briefly, first, cell lysates were harvested from both NAMPT-overexpressed MSCs and control MSCs. Isolated cell lysates were incubated with an anti-NAMPT antibody (Abcam, Cambridge, MA, USA ab236874) overnight at 4 °C. The next day, protein A agarose (Abcam, Cambridge, MA, USA ab193254) beads were added to the mixture to pull down NAMPT-immunoprecipitated cell lysates. The lysates were then incubated with substrates including ATP, nicotinamide, nicotinamide mononucleotide adenylyltransferase 1 (NMNAT1), and phosphoribosyl pyrophosphate (PRPP) at 30 °C for 30 min. After incubation, a mixture of water-soluble tetrazolium salts (WST-1), alcohol dehydrogenase (ADH), diaphorase, and ethanol were added to each sample to generate NAD^+^. The activity of NAMPT was then measured by determining the optical density (OD) at 450 nm using a microplate reader (Thermo Fisher Scientific, Waltham, MA, USA) every 5 min for 3 h in the dark.

### 4.6. Exosome Isolation and Characterization

Exosomes were isolated by differential ultracentrifugation as previously described [55]. Briefly, cells were cultured in an exosome-depleted FBS (Thermo Fisher Scientific, Waltham, MA, USA) containing medium, and once they reached 80–90% confluency, the conditioned culture medium was harvested for exosome isolation. First, the conditioned medium was centrifuged at 300× *g* for 10 min at 4 °C to remove cells and large debris, followed by centrifugation at 2000× *g* for 10 min at 4 °C to remove smaller cellular debris. The collected supernatant was further centrifuged at 10,000× *g* for 30 min to remove apoptotic bodies and large microvesicles. Next, the supernatant was passed through a 0.22 µm filter, and the filtrate was subjected to ultracentrifugation at 110,000× *g* for 70 min at 4 °C. The resultant supernatant was carefully discarded, and the pellet was resuspended in PBS and washed by a second ultracentrifugation at 110,000× *g* for 70 min. The final pellet was resuspended in sterile PBS. Isolated exosomes were detected using an exosome antibody array kit (Exo-Check, System Biosciences, Palo Alto, CA, USA) according to the manufacturer’s instructions. Cryo-transmission electron microscopy (cryo-TEM) was used to visualize the exosome particles. Total protein content was measured by bicinchoninic acid assay (BCA) using a BCA Protein Assay Kit (Thermo Fisher Scientific, Waltham, MA, USA). Isolated exosomes were aliquoted and stored at −80 °C until use.

### 4.7. Quantification of Exosome Particles

Exosome abundance was quantified using the FluoroCet Exosome Quantitation Kit (System Biosciences, Palo Alto, CA, USA) according to the manufacturer’s instructions. Briefly, for each reaction, 0.5 µg exosomal protein was mixed with the supplied lysis buffer and incubated for 30 min to liberate exosomal contents. A standard curve was prepared in parallel using the provided exosome standard, which is calibrated to known exosome amounts by nanoparticle tracking analysis. After lysis, the substrates were added and the mixture was incubated at room temperature in the dark for ~20 min. Fluorescence was measured using a microplate reader (excitation 530 nm, emission 590 nm). Background-subtracted fluorescence values were interpolated from the serially diluted standard curve to calculate the number of exosome particles in each sample.

### 4.8. Cryo-Transmission Electron Microscopy

Cryo-transmission electron microscopy (TEM) of isolated exosome samples was performed as previously described [56]. Briefly, A FEI Vitrobot (Mark IV) plunge freezer, set at room temperature and ~95% humidity, was used to prepare vitrified cryo-TEM specimens from the isolated exosome samples dissolved in PBS. About 2.5 µL of the solution was applied to a TEM grid coated with lacey carbon film. After blotting using two filter papers, the grid was plunge-frozen in liquid ethane. The vitrified specimen was mounted onto a Gatan 626.DH cryo-TEM holder and transferred into a FEI Tecnai F20 TEM (Thermo Fisher Scientific, Waltham, MA, USA) equipped with a Gatan twin blade retractable anti-contaminator. The cryo-TEM observation was carried out at 200 kV and ~−174 °C.

### 4.9. Exosome Treatment

EC CXCR4 KO (AS) mice were assessed by echocardiography for the development of aortic stenosis (AS) at 6 weeks of age, and mice that developed AS were randomly divided into three groups using a computer-generated sequence. AS mice were treated with intraperitoneal (IP) injection of 100 µL PBS (control group) and 100 µg exosomes (~1.91 × 10^11^ particles; suspended in 100 µL PBS) derived from either NAMPT-overexpressed MSC (NAMPT-Exo group) or regular MSC (MSC-Exo group) for three doses once a week. Follow-up echocardiography was performed at 7-day intervals after the first dose to monitor functional progression. Seven days after the final (3rd) dose of injection, endpoint echocardiography was performed, and thereafter, mice were harvested for histological and other assessments (Figure 3A).

### 4.10. Echocardiographic Measurements

Echocardiography was performed under anesthesia with 1.5–2% isoflurane using the VEVO 770 machine (FUJIFILM VisualSonics, Toronto, ON, Canada) at baseline before injection and weekly until sacrifice at 7 days post-endpoint injection. M-mode images and two-dimensional parasternal short-axis images at the mid-papillary level of each mouse were recorded. Aortic velocity, pressure, ejection fraction (EF%), fractional shortening (FS%), diastolic thicknesses of the LV posterior wall, systolic thicknesses of the LV posterior wall, diastolic LV internal dimensions (LVIDd), and systolic LV internal dimensions (LVIDs) were measured and calculated using VEVO LAB 3.0 software. Measurements were performed and analyzed by investigators who were blinded to the treatment and identity of the mouse.

### 4.11. Tissue Harvesting and Fixation

Mice were euthanized after endpoint echocardiography to harvest the heart and other tissue samples. The harvested hearts were sectioned into thirds using a heart matrice (Braintree, #BS-SS-H 5005, Chicago, IL, USA). The bases containing the aortic valves were fixed in 10% Neutral Buffered Formalin (Fisher, Waltham, MA, USA, #22-110-683) overnight at room temperature. The apex and midsection of the heart were snap-frozen and stored at −80 °C for later use. Fixed tissue sections were then processed, paraffin-embedded, and sectioned with 5 um thickness using a Leica microtome.

### 4.12. Histological Analysis

Hematoxylin and Eosin (H&E) staining was performed for general morphology. Base samples containing the aortic valve were stained using Alizarin Red (Sigma, #A5533, Burlington, MA, USA) for the detection of microcalcification. Staining sections were imaged using a slide scanner (Olympus BX61VS, Webster, TX, USA) at 40× magnification. Quantification of calcification was performed using ImageJ 1.53 software by observers blinded to treatment groups.

### 4.13. Immunohistochemistry

After deparaffinization and rehydration, the aortic valve sections were boiled at 100 °C in Tris-EDTA buffer (pH 9.0) for 15 min to facilitate antigen retrieval. Once cooled to room temperature, the sections were incubated with blocking buffer (1% bovine serum albumin) for 45 min at room temperature, followed by overnight incubations with primary antibodies at 4 °C. The primary antibodies used are rhodamine-conjugated WGA (Vector Laboratories, Newark, CA, USA, RL1022, 1:250), tdTomato (ORIGENE, Rockville, MD, USA, AB8181200, 1:200), Griffonia Simplicifolia Lectin I Isolectin B4 Fluorescein (Vector Laboratories, Newark, CA, USA, FL-1201, 1:50) and Alpha-SMA (Thermo Fisher Scientific, Waltham, MA, USA, PA5-85919, 1:200). After washing, the sections were incubated with the secondary antibodies at room temperature for 1 h. The secondary antibodies used are Rabbit anti-Goat IgG, Alexa Fluor^TM^ 488 (Thermo Fisher Scientific, Waltham, MA, USA, A11078, 1:250) and Goat anti-Rabbit IgG, Alexa Fluor^TM^ 594 (Thermo Fisher Scientific, Waltham, MA, USA, A11037, 1:250). Sections were mounted with a mounting media containing DAPI (Prolong Diamond Antifade Mountant with DAPI-Thermo Fisher Scientific, Waltham, MA, USA, P36971) to counterstain nucleus and were imaged with a confocal microscope (name). All quantitative analyses were performed with ImageJ 1.53 software.

### 4.14. Endothelial Cell Isolation and Culture

Mouse cardiac endothelial cells (ECs) were isolated as previously described [26]. Briefly, after dissecting and mincing the hearts into small pieces, Collagenase I (Worthington, Lakewood, NJ, USA) was used to digest the tissues. After washing, digested cells were incubated with Dynabeads conjugated with anti-CD31 antibody (Thermo Fisher Scientific, Oakwood, OH, USA). The beads with endothelial cell mixtures were washed several times and cultured in a mouse endothelial culture medium (Cell Biologics, Chicago, IL, USA). Once the cells reached confluency, cells were purified with Dynabeads conjugated with anti-Mouse CD102 (ICAM2) antibody.

### 4.15. EndMT Assay

EndMT assay was performed as previously described [36]. Briefly, isolated cardiac endothelial cells were plated at 2.5 × 10^3^ cells/cm^2^ for 24–48 h. before exposing them to various treatment groups: Control (no treatment), TGF-β1 (recombinant mouse TGF-β1, 10 ng/mL, R&D Systems, Minneapolis, MN, USA, 7666-MB-005), and TGF-β1+miR-146a-3p inhibitor/miR NC5 negative control (Integrated DNA Technologies, Coralville, IA, USA). MiR-146a-3p and miR NC5 negative control sequences are listed in Table 2.

Cells were treated with TGF-β1 for 3 days in the presence or absence of miR-146a-3p inhibitor/miR NC5 negative control. In the TGF-β1+miR-146a-3p inhibitor/miR NC5 negative control experimental group, cells were transfected with either the miR-146a-3p inhibitor or the miR NC5 negative control using a transfection reagent (Lipofectamine RNAiMAX, Thermo Fisher Scientific, Waltham, MA, USA) according to the manufacturer’s instructions 24 h before the start of TGF-β1 treatment. Cell morphology was monitored regularly, and after the end of the treatment, cells were harvested either for immunofluorescence study or Western blot.

### 4.16. Immunocytochemistry and Immunofluorescence Staining

Cardiac endothelial cells, either untreated or treated with TGF-β1 in the presence or absence of the miR-146a-3p inhibitor, were fixed in −20 °C methanol for 10 min. After fixing, they were blocked with 3% bovine serum albumin for 45 min at room temperature, followed by incubation with primary antibodies CD31 (Proteintech, Rosemont, IL, USA, 66065-2-Ig, 1:1000) and Alpha-SMA (Thermo Fisher Scientific, Waltham, MA, USA, PA5-85919, 1:200) overnight at 4 °C in the dark. The next day, after washing, the cells were incubated with corresponding fluorescent secondary antibodies, Coralite 488-conjugated goat anti-mouse IgG (Proteintech, Rosemont, IL, USA, SA00013-1, 1:200) and goat anti-rabbit IgG, Alexa FluorTM 594 (Thermo Fisher Scientific, Waltham, MA, USA, A11037, 1:250) at room temperature for 1 h. After washing, slides containing the immunolabeled cells were mounted with a mounting medium containing DAPI (Prolong Diamond Antifade Mountant with DAPI-Thermo Fisher Scientific, Waltham, MA, USA, P36971). Images were obtained using a confocal microscope, and all quantitative analyses were performed with ImageJ 1.53 software (NIH website) by investigators who were blinded to group allocation. All experiments were repeated at least two times, and representative results are shown.

### 4.17. Western Blot Analysis

Exosomes, tissues, or cells were lysed with a RIPA Kit (Sigma Aldrich, Waltham, MA, USA, R0278) supplemented with protease and phosphatase inhibitors (Thermo Fisher Scientific, Waltham, MA, USA, 78440). The extracted protein concentration was determined using the BCA protein assay (Thermo Fisher Scientific, Waltham, MA, USA, 23227) according to the manufacturer’s instructions. The extracted protein was subjected to SDS-PAGE electrophoresis separation and then transferred to polyvinylidene fluoride membranes. After that, the membranes were blocked either with 5% milk or BSA for 1 h to prevent nonspecific binding and then incubated overnight with primary antibodies at 4 °C. The primary antibodies used are NAMPT (Proteintech, Rosemont, IL, USA, 11776-1-AP, 1:4000), CD31 (Bioss, Woburn, MA, USA BS-0468R, 1:500), VE Cadherin (Thermo Fisher Scientific, Waltham, MA, USA, 36-1900, 1:250), Alpha-SMA (Thermo Fisher Scientific, Waltham, MA, USA, PA5-85919, 1:2000), Vimentin (Proteintech, Rosemont, IL, USA, 10366-1-AP, 1:20000), GAPDH (Proteintech, Rosemont, IL, USA, 60004-1-Ig, 1:30000), and Beta-Actin (Cell Signaling, Danvers, MA, USA, 4970, 1:1000). Following primary antibody incubation, the membranes were washed and were incubated with secondary antibodies for 1 h at room temperature. The secondary antibodies used are Goat anti-rabbit IgG, HRP (Cell Signaling, Danvers, MA, USA, 7074, 1:2000) and Goat-anti-mouse IgG2b, HRP (Thermo Fisher Scientific, Waltham, MA, USA, M32407, 1:4000). Immunoreactive bands were detected using a Western blot imaging system (Cytiva, Marlborough, MA, USA, Amersham ImageQuant 800). Image analysis and blot quantification were performed with ImageJ 1.53 software.

### 4.18. MicroRNA qPCR

Total RNA was extracted from cardiac endothelial cells using Trizol (Invitrogen, USA). Polyadenylation and reverse transcription were performed using the Mir-X™ miRNA First Strand Synthesis Kit (Takara Bio, Kusatsu, Shiga, Japan), and the TB Green kit (Takara Bio, Kusatsu, Shiga, Japan) was used to quantify the miRNA expression level using real-time polymerase chain reaction. Relative gene expression was calculated using the 2(−ddCt) method with U6 (Takara Bio, Kusatsu, Shiga, Japan) as the housekeeping gene. Primer sequences used are listed in Table 3.

### 4.19. Data Preprocessing and Quality Control

RNA concentration and purity were assessed using a NanoDrop spectrophotometer, and samples with A260/280 ratios between 1.8 and 2.0 were included for downstream analyses. RNA integrity was further verified by electrophoretic profiling when sufficient material was available. MicroRNA expression was measured by RT–qPCR using miRNA-specific primers in technical triplicates with appropriate negative controls. Ct values were normalized to U6 as an internal control, and relative expression was calculated using the 2^−ΔΔCt^ method with CXCR4^fl/fl^ controls set to 1.0.

### 4.20. Study Power and Sample Size

Sample size was determined based on prior similar studies to ensure adequate power to detect biologically relevant differences. All analyses were conducted according to predefined group sizes established prior to experimentation.

### 4.21. Statistical Analysis

All data are expressed as mean  ±  standard deviation (SD). Comparisons between two groups were assessed by the Student *t*-test. One-way ANOVA followed by post hoc Tukey (when every mean was compared with every other mean) or Dunnett test (when the means of different experimental groups were compared against a control mean) was performed to compare among more than two groups. Statistical analyses were performed using GraphPad Prism 9. A *p*-value of <0.05 (two-sided) was used to establish statistical significance.

## 5. Conclusions

In summary, our study demonstrates that NAMPT-enriched MSC-derived exosomes effectively slow aortic stenosis progression in EC CXCR4 KO mice, improving valvular hemodynamics and cardiac function. These effects are mediated, at least in part, by suppression of EndMT and normalization of miR-146a-3p expression. Our findings highlight exosome-based delivery of targeted molecular cargo as a promising non-invasive strategy for AS, offering potential to modify disease progression and delay or reduce the need for valve replacement.

## Figures and Tables

**Table 1:** Primer sequences used for NAMPT amplification.

Primer Type	Sequence (5′-3′)	Restriction Site
Forward primer	CGGTGAATTCCTCGAGATGAATGCTGCGGCAGAAGC	XhoI
Reverse primer	GAGAGGGGCGGGATCCCTAATGAGGTGCCACGTCCTG	BamHI

**Table 2:** MiRNA sequences used for EndMT assay.

miRNA	Sequence (5′-3′)
miR-146a-3p	CCUGUGAAAUUCAGUUCUUCAG
miR-NC5 negative control	ACCAUAUUGCGCGUAUAGUCGC

**Table 3:** Primer sequences used for miRNA quantification.

miRNA	Sequence (5′-3′)
mmu-miR-146a-5P	UGAGAACUGAAUUCCAUGGGUU
mmu-miR-146a-3P	CCUGUGAAAUUCAGUUCUUCAG
mmu-miR-142a-5P	CAUAAAGUAGAAAGCACUACU
mmu-miR-125b-5P	UCCCUGAGACCCUAACUUGUGA

## Data Availability

The data presented in this study are openly available in DRYAD at http://datadryad.org/share/LINK_NOT_FOR_PUBLICATION/vOoJ0W_Y4FHVzKiRur_gfxTbLgQCklhO8eak80H4BC4 (accessed on 8 October 2025).

## Abbreviations

AS	Aortic stenosis
AV	Aortic Valve
BCA	Bicinchoninic acid
CD31	cluster of differentiation 31
CXCR4	C-X-C chemokine receptor type 4
DAPI	4′,6-Diamidino-2-phenylindole
EC	Endothelial cell
EC-CXCR4 KO	Endothelial cell-specific CXCR4 knockout
EndMT	Endothelial-to-mesenchymal transition
EV	Extracellular Vesicles
FBS	Fetal bovine serum
H&E	Hematoxylin and eosin
IP	Intraperitoneal
LVID	Left Ventricular internal diameter
miRNA	microRNA
MSC	Mesenchymal stem cell
NAD	Nicotinamide adenine dinucleotide
NAMPT	Nicotinamide phosphoribosyltransferase
NAMPT-Exo	NAMPT-enriched mesenchymal stem cell-derived exosomes
qPCR	Quantitative polymerase chain reaction
RT	Room temperature
TEM	Transmission electron microscopy
TGF-β1	Transforming growth factor-beta 1
TSG101	Tumor susceptibility gene 101
WTA	Wheat Germ Agglutinin
α-SMA	α-smooth muscle actin
